# Carbon Monoxide Promotes Respiratory Hemoproteins Iron Reduction Using Peroxides as Electron Donors

**DOI:** 10.1371/journal.pone.0033039

**Published:** 2012-03-12

**Authors:** Elena A. Sher, Mati Shaklai, Nurith Shaklai

**Affiliations:** 1 Department of Human Molecular Genetics and Biochemistry, Sackler Faculty of Medicine, Tel-Aviv University, Tel Aviv, Israel; 2 Department of Hematology, Sackler Faculty of Medicine, Tel-Aviv University, Tel Aviv, Israel; Consejo Superior de Investigaciones Cientificas, Spain

## Abstract

The physiological role of the respiratory hemoproteins (RH), hemoglobin and myoglobin, is to deliver O_2_ via its binding to their ferrous (Fe^II^) heme-iron. Under variety of pathological conditions RH proteins leak to blood plasma and oxidized to ferric (Fe^III^, met) forms becoming the source of oxidative vascular damage. However, recent studies have indicated that both metRH and peroxides induce Heme Oxygenase (HO) enzyme producing carbon monoxide (CO). The gas has an extremely high affinity for the ferrous heme-iron and is known to reduce ferric hemoproteins in the presence of suitable electron donors. We hypothesized that under *in vivo* plasma conditions, peroxides at low concentration can assist the reduction of metRH in presence of CO. The effect of CO on interaction of metRH with hydrophilic or hydrophobic peroxides was analyzed by following Soret and visible light absorption changes in reaction mixtures. It was found that under anaerobic conditions and low concentrations of RH and peroxides mimicking plasma conditions, peroxides served as electron donors and RH were reduced to their ferrous carboxy forms. The reaction rates were dependent on CO as well as peroxide concentrations. These results demonstrate that oxidative activity of acellular ferric RH and peroxides may be amended by CO turning on the reducing potential of peroxides and facilitating the formation of redox-inactive carboxyRH. Our data suggest the possible role of HO/CO in protection of vascular system from oxidative damage.

## Introduction

Hemoglobin (Hb) and myoglobin (Mb) are collectively known as the respiratory hemoproteins (RH) based on their function to deliver molecular oxygen to the body tissues. While the link between ferrous (Fe^II^) heme-iron and molecular oxygen provides the essence of aerobic metabolism, this molecular function is also vulnerable to detrimental effects by auto-oxidation due to electron transfer from the heme-iron to oxygen: Fe^II^-O_2_→Fe^III^+O^·^_2_→H_2_O_2_ [1]. The products of the reaction, ferric (Fe^III^, met-) RH as well as active oxygen species, are hazardous to the vascular system, as they can induce pathological events, such as atherosclerosis. The ferric forms of the RH bear peroxidase-like activity leading to the unstable, potentially damaging ferryl (Fe^IV^) state [2]–[5], [6], [7]. The ferryl forms tend to undergo synproportionation in presence of ferrous, oxy-forms to the more stable ferric forms (Fe^II^+Fe^IV^→2Fe^III^) which is longer-lived and hence its likelihood to provide damage are high [8]. Indeed, under a variety of oxidative pathological conditions, ferric RH have been reported to be elevated in blood plasma [9]–[12].

The pathological redox interactions of metRH with hydrophilic peroxides, i.e. H_2_O_2_, have been elucidated over the years and their reaction mechanisms have been analyzed in detail on the molecular level [2], [13], [14]. Interactions between the hydrophilic hemoproteins and hydrophobic peroxides, mostly formed in cell membranes and plasma lipoproteins, i.e. LDL, have been documented as well [15]–[17]. A wealth of information regarding the RH interactions with LDL, induced by both H_2_O_2_ and hydrophobic peroxides within the LDL particles has been reported [15], [18], [19]. Peroxides acquired their notorious reputation as damaging oxidative agents, especially upon interaction with RH [3]. It should be emphasized that damage by intact metRH forms can be attenuated as long as the hemin is globin bound, namely metRH function as intact heme-proteins. Slow irreversible detachment of hemin from ferric RH occurs as well and its hazardous activity is irreversible [15]. An evolutionary advantage was gained by encapsulating the RH in erythrocytes or myocytes which contain a collection of enzymes responsible for reducing any ferric heme formed back to its ferrous state [20], [21]. After multiple trials, the tendency of cell-free hemoglobin to undergo undesired oxidations has so far impeded efforts to use stabilized hemoglobin solutions as blood substitutes [22]. It is important to note that peroxides can also function as reducing agents [23]. In fact, in the plant kingdom, peroxides are active as electron donors, namely as reducing agents [24]. Interestingly, current studies have pointed out that under certain conditions, cell-free hemoglobin also acts as an antioxidant and protects different cell types from H_2_O_2_-induced oxidative damage. It was thus proposed that complex interactions determine whether Hb will act as a damaging or protective agent under different circumstances, the mechanisms behind this dual chemical nature are still obscure [25].

Although the RH function primarily as oxygen carriers, lately attention has been focused on their role as physiological carriers of two other gas ligand molecules, nitric oxide (NO), which binds to ferrous as well as to ferric heme-iron, and carbon monoxide (CO), which binds with extremely high affinity exclusively to ferrous heme-iron [26]–[30]. The latter gases were considered for years to be toxic molecules based on their competition with oxygen for the heme-iron. Yet, once it became clear that NO is involved in a variety of metabolic signaling pathways, its recognition as a physiological regulator in vascular and other organ functions became established [28], [31].

In recent years the essential role of CO, the product of heme catabolism by the enzyme Heme Oxygenase (HO, specifically its inducible form, HO-1) has been clarified [32]–[35]. While it is obvious that HO's protective function relates to heme clearance, this enzyme was reported also to be involved directly and indirectly in a variety of central protective physiologic mechanisms, many of which are mediated by its product, CO [29], [30], [36]–[38]. Specifically, progression of atherosclerosis is effectively inhibited by upregulation of HO-1/CO [39]. The specific high affinity of CO for ferrous iron calls for its broad optional involvement in hemoproteins reduction. Indeed, previous studies have indicated CO-driven ferric heme reduction. One such reduction mechanism is built-in as in the enzyme cytochrome C oxidase, where existence of copper in porphyrin aids electron transfer, as the enzyme function is to reduce oxygen [40], [41]. In contrast, in the case of a respiratory protein like hemoglobin, which lacks built-in electron transfer assistance, an external reducing agent is required. We hypothesized that under *in vivo* conditions peroxides in low concentration can assist the reduction in presence of CO.

The present study was designed to analyze *in vitro* on a molecular level, whether the potential dangerous oxidative activities of acellular RH and peroxides can be attenuated by the HO product, CO. To mimic *in vivo* plasma conditions, reactions were carried out in an anaerobic environment using ferric RH and peroxides at their concentration levels reported under a variety of pathological circumstances. It was found that in the presence of CO damaging peroxides are consumed while ferric RH are transformed into their nonhazardous carboxy-forms.

## Results

### Effect of CO on the interaction of hydrophilic (H_2_O_2_) peroxides with ferric hemoproteins

**Figure 1:**
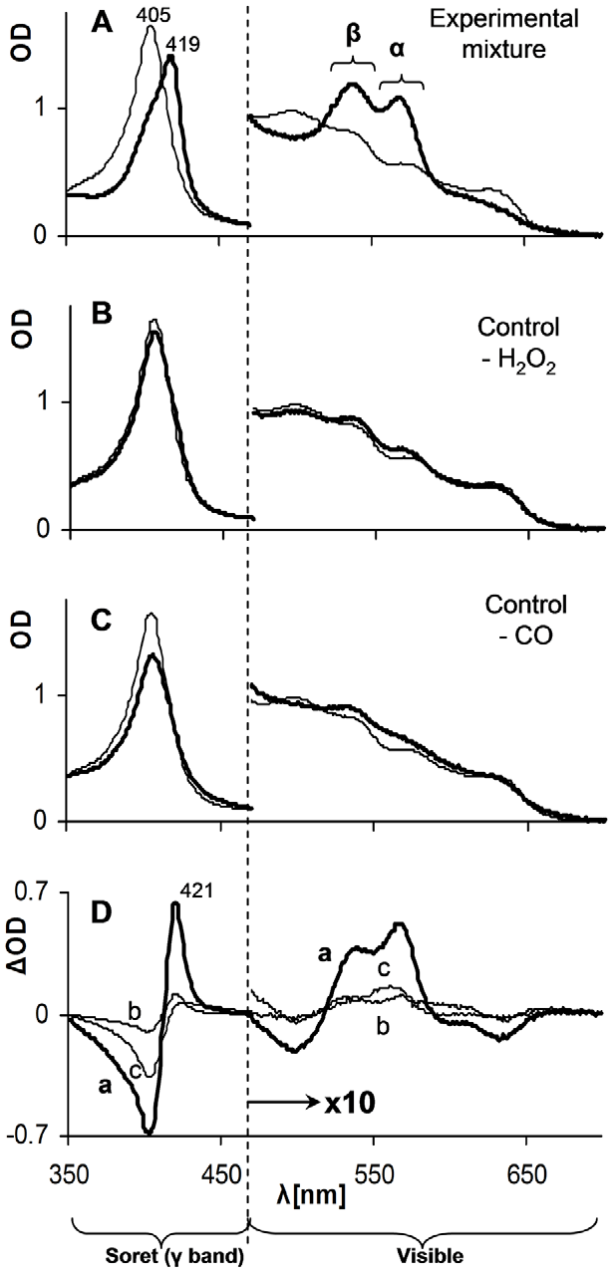
Effect of CO atmosphere on the interaction of H_2_O_2_ with ferric Hb. Light absorption spectra of the reaction mixtures at time zero (narrow lines) and following two hours of incubation (heavy lines) are depicted. **A** – experimental mixture containing ferric Hb and H_2_0_2_ under CO atmosphere (∼1 mM in water phase); **B** – control reaction mixture without H_2_O_2_; **C** – control reaction mixture in which CO atmosphere was replaced by inert gas; **D** – difference spectra (ΔOD) of time zero and following 2 hours of A, B and C (marked by small letters a, b and c). Note scale differences of Soret and visible regions.

The first part of the study was designed to test whether CO can affect the interaction between metHb (Fe ^III^) and H_2_O_2_. To mimic *in vivo* conditions of restricted free oxygen (under physiological condition, practically all oxygen is hemoglobin bound) the reactions were carried out under oxygen-free conditions in a CO atmosphere which resulted in a CO concentration of 1 mM in water phase [42], [43]. Hb and H_2_O_2_ were used at a concentration range of few micromolars throughout the study. Control reactions were carried out under neutral gases, argon or nitrogen, without CO or in a CO atmosphere in which no peroxides were added. The data in Figure 1A indicate that within two hours significant spectral changes occurred in the experimental mixture: red shift of the Soret (γ, 405–440 nm) band and the formation of two new prominent peaks in the visible region bands (α, ∼570 nm and β, ∼540 nm). The newly formed spectrum (Fig. 1A, bold line) resembles that of the well characterized carboxyHb (COHb, Soret peak at 419 nm, α band at 569 and β band at ∼540 nm) [44]. In contrast, in control mixtures lacking either CO or H_2_O_2_ practically no changes occurred in the spectra pattern and only minor changes in the optical density (OD) were detected (Fig. 1B & C). Similar results were observed by replacing Hb by Mb (data not shown).

The spectral changes are easier noticeable when presented as difference spectra depicted in Figure 1D. The minimal alterations in the absence of CO (line c) result from ferryl formation and hemin oxidative disintegrations by H_2_O_2_ as discussed in the literature [2], [3]. In absence of H_2_O_2_ (line b) spectral changes, due to the reduction of metHb under CO atmosphere lacking a reducing agent, have already been described, however they are extremely slow (t_1/2_ of ∼50 days) [45].

**Figure 2:**
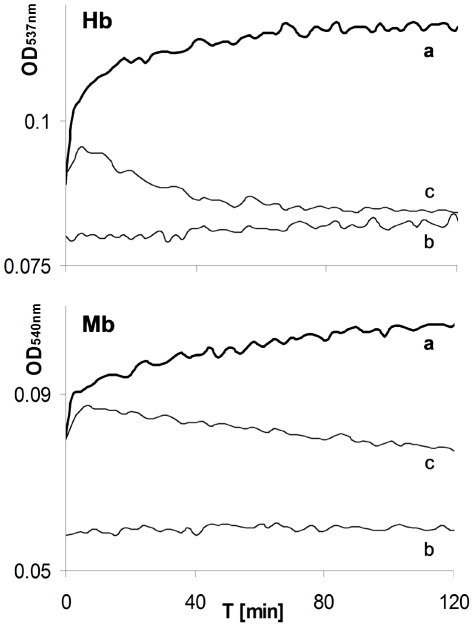
Effect of CO on time dependent patterns of carboxyRH forms β band (∼540 nm). The “a” curves in each panel depict experimental mixtures containing ferric Hb (upper panel) or Mb (lower panel) and H_2_0_2_ in CO atmosphere. The “b” curves in each panel depict controls lacking H_2_O_2_. The “c” curves in each panel depict controls in which the CO atmosphere was replaced by an inert gas. (Time zero = ∼1 min following mixing.).

Time dependent spectral changes of reactions including Hb or Mb (Fig. 2) at wavelengths of carboxy β bands (∼540 nm) indicated that all H_2_O_2_-induced alterations were completed within two hours. The kinetic patterns of the CO lacking controls of both hemoproteins exhibited an initially fast absorption increase, followed by a decreasing phase (Fig. 2, lines c). Such a pattern is typical of ferryl (Fe^IV^) formation followed by its auto-reduction to ferric (Fe^III^) forms [45]. In peroxide lacking controls only moderate increases in absorption could be observed (Fig. 2, lines b) which correlated with Figure 1 B &D (line b) data and previous literature [45]. In contrast, in the experimental reaction mixtures, only increased absorption occurred (Fig. 2, lines a). These kinetic patterns provide additional evidence that in the presence of CO, the stable carboxy-hemoproteins, rather than the unstable ferryl forms, were produced. Consistent with the above conclusion, addition of sodium sulfide (Na_2_S) two hours after reaction initiation did not result in any formation of the typical ferryl product (data not shown) [46]. Using ΔOD of the α band (∼570 nm, Fig. 1A) and the related extinction coefficient, the amount of COHb formed was estimated at least 50% of initial Hb content despite partial heme disintegration [47]. To eliminate hemin disintegration and significant peroxidase-like contribution, in the next experiment concentrations of H_2_O_2_ used were equal or lower then those of Hb.

**Figure 3:**
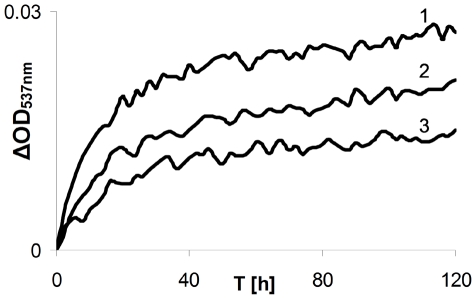
Dependence of carboxyHb formation rate on H_2_O_2_ concentration. Shown are the β band (537 nm) time dependent patterns of experimental mixtures containing ferric Hb in CO atmosphere following an addition of H_2_0_2_ in concentrations of: 10 µM - curve 1, 5 µM - curve 2, 2.5 µM - curve 3.

The results demonstrated in Figure 3 indicate that the rates of metHb reduction and COHb formation as well as final amount of COHb produced within 2 hours are H_2_O_2_ concentration dependent.

**Figure 4:**
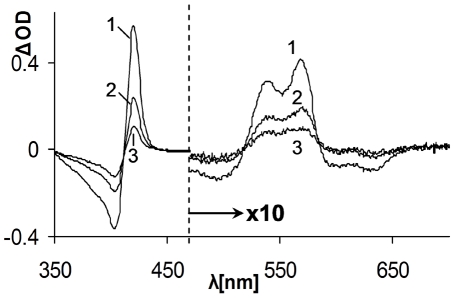
Interaction of low CO concentration with H_2_O_2_/ferric Hb. Reaction mixtures contained ferric Hb, H_2_O_2_ and differing concentrations of CO in the experimental environment. Difference spectra of time zero and ON incubation after subtraction of CO lacking control are presented. Curve 1 – 1 mM CO in reaction mixture (100% CO in atmosphere); Curve 2 - 0.2 mM CO in reaction mixture (CO 20%/N_2_ 80% in atmosphere); Curve 3 – 0.05 mM Co (CO5%/N_2_ 95% in atmosphere). Note scale differences of Soret and visible regions.

The experiments up to this stage were performed in solutions equilibrated with an atmosphere containing CO alone which, as stated, yield about 1 mM of the gas in the liquid phase. Such a concentration is not expected to be formed in the plasma even following long-term activated HO-1. Therefore the following experiment tested whether H_2_O_2_ can be effective as a ferric Hb reducing agent in the presence of lower CO concentrations. Free oxygen was also avoided in this experiment by using a neutral gas (nitrogen) as a complement. As slower reaction rates are expected by lowering one of reactant concentrations, the reaction mixtures were incubated overnight (ON). Figure 4 depicts difference absorption spectra following the incubation period. As can be seen, at lower CO concentrations ferric to carboxyHb transformation occurred as well.

### Effect of CO on the interaction of hydrophobic (lipid) peroxides with ferric hemoproteins

**Figure 5:**
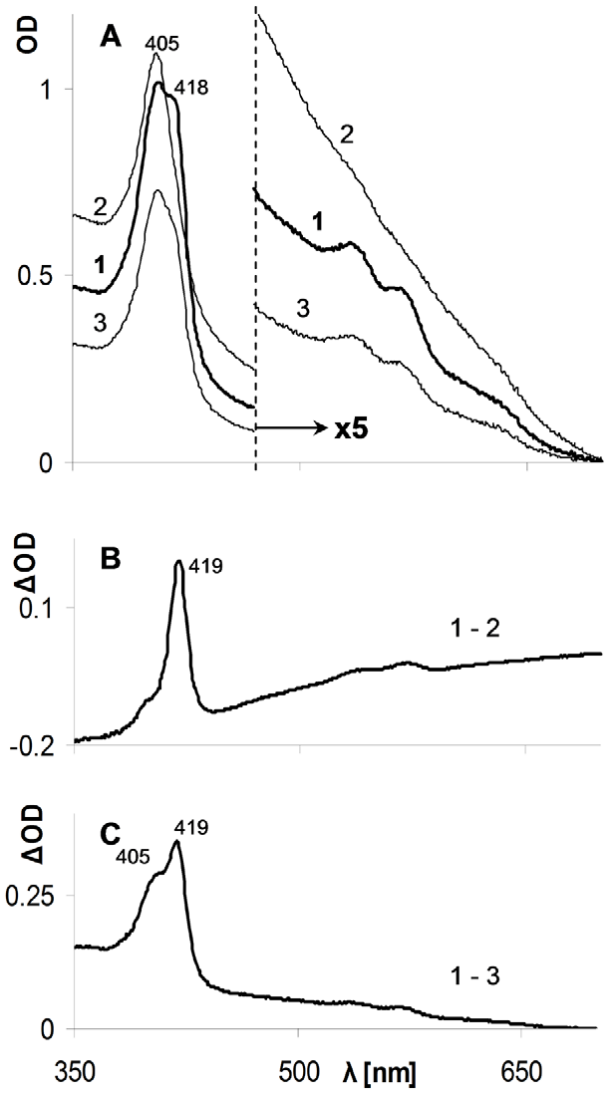
Effect of CO on the interaction of ferric Hb with copper oxidized LDL. **A** - Spectra of reaction mixtures incubated ON: Curve 1 - experimental mixture containing ferric Hb, oxLDL and 1 mM CO; Curve 2 – control in which CO was replaced by inert gas in the experimental environment; Curve 3 - control in which oxLDL was replaced by nLDL. Note scale differences of Soret and visible regions. **B** - Difference spectrum of curves 1 & 2 in A. **C** - Difference spectrum of curves 1 & 3 in A.

To analyze a possible interaction of hydrophobic peroxides and metHb, oxidized LDL (oxLDL) formed by copper were used initially [48]. The experimental mixture containing ferric Hb and oxLDL was incubated under CO atmosphere. For this reaction, two types of controls were applied: the same mixture incubated under neutral gas (argon) or replacement of oxLDL by fresh, native, peroxide-poor LDL (nLDL). Figure 5 illustrates the original and difference spectra of the experimental and control mixtures following ON incubation. Comparison of the samples kept under CO (line 1) and inert gas (argon, line 2) indicates clear differences: in the reaction mixture incubated under inert gas, the increase in OD is strictly a result of light scatter, since no red shift of the Soret (γ) band, nor formation of new peaks in the visible region bands were observed. In contrast, the absorption spectrum of the sample incubated under CO demonstrates two new spectral features: (a) formation of peaks in the visible spectrum; (b) formation of a doublet Soret peak. The double Soret peak results from part of the hemoprotein shifting to the red. The difference spectrum of 1&2 (Fig. 5B) resembles a typical COHb spectrum and hence indicates the formation of this adduct. It is well documented that ferric Hb causes LDL oxidation followed by apoB protein cross-linking which produces light scattering aggregates [7], [15], [48], [49]. Thus, the high light scatter observed under the inert gas (Fig. 5A, line 2) points to increased LDL oxidation in this sample as compared to the reaction under CO atmosphere (Fig. 5A, line 1). Regarding the nLDL control (Fig. 5A, line 3), some COHb is formed as well, probably by the involvement of low level peroxides existing even in fresh LDL [50]. On the other hand, the difference absorption spectrum in Figure 5C clearly indicates that when LDL is loaded with peroxides (oxLDL), more COHb is formed. Moreover, the Soret double peak (405 & 419 nm) in the difference spectrum indicates, that the oxLDL sample contains a higher level of residual ferric Hb.

**Figure 6:**
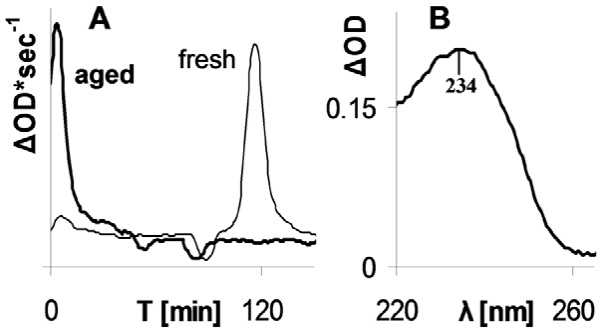
Increased peroxidation in aged LDL. **A** - Oxidation by hemin (1 µM) presented as derivatives of time course (λ_268 nm_), indicating formation of conjugated dienes. Heavy line – aged LDL, narrow line – fresh LDL. **B** - Increased content of conjugated dienes in aged LDL presented as difference spectrum of aged and fresh LDL.

Having demonstrated that the copper oxidized LDL model can replace hydrophilic peroxides as reducing agents for metHb in presence of CO, it was of importance to study the activity of “naturally” oxidized aged LDL which can form *in vivo* [48]. Aged LDL was produced from a freshly isolated sample by long term incubation (see methods) [51]. To evaluate whether aged LDL contained a higher level of peroxides, their oxidizing potential was compared to that of fresh LDL from the same donor by standard parameters, oxidation lag time and level of conjugated dienes [52]. To measure the oxidation lag time, a minor amount of free hemin was used (see methods section). While fresh LDL had a lag time of about two hours, aged LDL had practically zero lag time (Fig. 6A). In addition, aged LDL, as compared to fresh one, demonstrated increased absorption at 234 nm indicating extra conjugated dienes (Fig. 6B).

**Figure 7:**
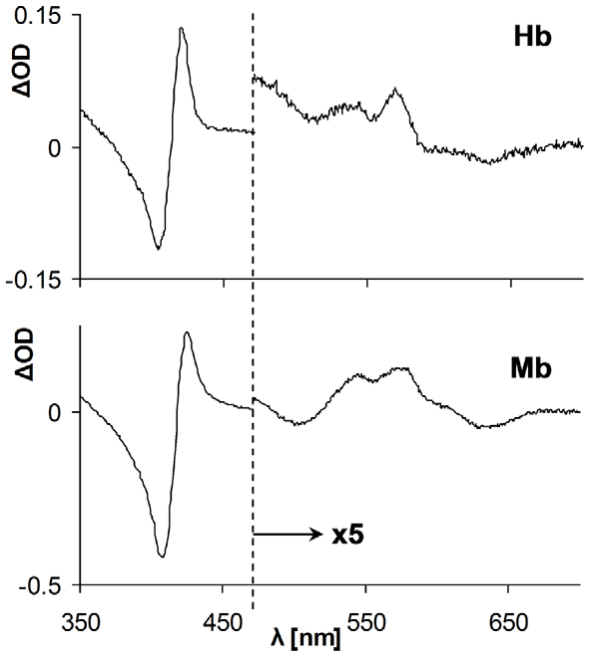
Effect of CO on the interaction of ferric RH with hydrophobic peroxides in aged LDL. Difference absorption spectra of time zero and ON incubated reaction mixtures containing ferric Hb (upper panel) or Mb (lower panel), aged LDL and 1 mM CO are shown. The spectrum of the LDL lacking control was subtracted from both reaction mixtures spectra. Note scale differences of Soret and visible regions.

Once the increased oxidability of aged LDL was demonstrated, it was further employed as a source of hydrophobic peroxides to determine whether they are able to act as reducers of ferric RH in presence of CO. Experimental mixtures containing ferric Hb or Mb and aged LDL were incubated under CO atmosphere. LDL lacking mixtures were used as controls. The difference spectra of time zero and ON incubation are depicted in Figure 7. The results revealed that although negligible amounts of carboxyRH formed in the LDL lacking controls, a considerably increased amount of COHb/Mb formed in the aged LDL containing reaction mixtures. These data clearly indicate that lipid peroxides react similarly to H_2_O_2_ as ferric heme-iron reducing agents and trap the RH in their carboxy, inactive forms.

## Discussion

The focus of the current study was to highlight the potential involvement of the HO-1, specifically its product CO, in limiting vascular oxidative stress mediated by cell-free RH. Our data showed that the combined presence of H_2_O_2_ and CO resulted in transformation of ferric RH into their carboxy forms (Fig. 1, 2, 3, 4). These results clearly showed the reduction of ferric heme-iron in the RH to ferrous state. The only source of reducing equivalents in this experimental system is the participating peroxide. Despite the weak reducing activity of peroxide, the fast and practically irreversible reaction of ferrous heme-iron with CO resulted in a shift of the equilibrium towards the carboxy forms of RH [53]. The two reaction steps and their relative rates based on the literature are summarized below:

1. H_2_O_2_+RH(Fe^III^)→RH(Fe^II^)+½O_2_+H_2_O (very slow)
2. RH(Fe^II^)+CO→CORH(Fe^II^) (fast)

As can be seen, the products of reaction I are molecular oxygen and water, obviously physiologically harmless components. CarboxyRH, the product of reaction II, is extremely stable, thus redox inactive. It appears therefore that the main impact of CO is not reduction of ferric heme-iron per se, but rather its arrest in the ferrous carboxy complex, a practically irreversible process. Equilibrium is then shifted via Le Chatelier's principle. Therefore, the net result of the reaction appears to be replacement of injurious plasma components, metRH and H_2_O_2_, by physiological, harmless, metabolites.

**Figure 8:**
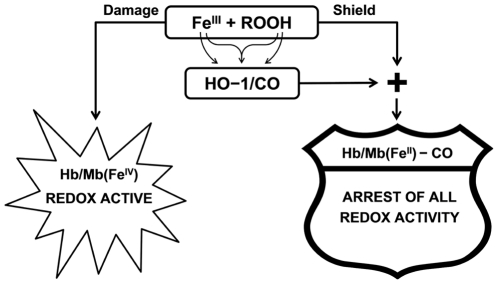
Schematic view of HO-1/CO protection mechanism. Left side: Damage induced by peroxides and peroxidase-like activity of ferric RH Right side: HO-1/CO shield provided by shifting the peroxide oxidation towards reduction thereby producing redox-inactive ferrous carboxyRH.

The link between oxidative conditions, elevated peroxides (hydrophilic and hydrophobic) and Hb in vascular pathology is well established [54]. Also both metHb and peroxides, readily formed under oxidative conditions, were proven to be inducers of HO-1 [55], [56]. The combined results establish the protecting role of HO-1 and its products as antagonists of both peroxide and heme-iron oxidative reactivity. These pathways are schematically illustrated in Figure 8.

It is important to note that other electron donors can affect ferric RH in a similar manner. For example, in presence of CO the reduction of ferric to ferrous Mb occurs in a mutant protein where a correctly positioned cysteine residue is responsible for electron transfer to the heme-iron [57]. Moreover, the combined effect of HO/CO system and peroxides to achieve the double purpose redox neutralization of damaging both peroxides and metalloproteins in the living kingdom is even beyond animal space. Several studies indicated that elevated HO or CO in plants result in decreased peroxides levels and lipid peroxidation [58].

Epidemiological studies evaluating metHb and COHb levels usually refer to total blood containing mostly encapsulated Hb. Since cell-free Hb is a minor part of total Hb, changes in this fraction are usually not observed. Interestingly, support for our *in vitro* data could be found from *in vivo* total hemoglobin evaluation following abdominal surgery [59]. Measurements were performed immediately post-operation and one day later. Although measurements were carried out in whole blood, careful examination of the results ([59] table 5) indicates that parallel increased COHb and decreased metHb were found only in those patients who did not receive blood transfusion one day after the operation which possibly reflect on much larger differences in cell-free in plasma Hb.

It should be noted that diminished oxidability of RH by CO using any electron source, even a weak one like peroxides, probably represents a much broader phenomena than that shown in the current study for the RH and peroxides. Horseradish Peroxidase (HRP) is a classic heme-enzyme with a ferric ground state is also reduced to ferrous iron thereby binding CO with high affinity [60]. HRP efficiently uses peroxides to oxidize diverse substrates including LDL. It appears that ferric-HRP can be reduced by LDL peroxides and thus arrested in a CO bound ferrous state [50]. The difference between a classic hemo-peroxidase and pseudo RH peroxidases is that while the ferric state of the classic enzyme is stable, that of RH specifically Hb, can transfer its hemin to blood components like LDL where an far-reaching oxidation takes place [15]. However shutting down any electron transfer from the heme iron by CO will arrest both pathways.

An additional aspect related to the consequences of the present study involves iron chelators. These molecules, originally designed to strongly bind hazardous free iron and eliminate it from plasma, were recently demonstrated to function as antioxidants as well. By binding to ferric heme-iron, the chelators prevent also oxidative modifications [61]. In the presence of CO, the oxidation is actually prevented by a similar mechanism, only that in this case the heme-iron is trapped in its ferrous state.

As stated, the current *in vitro* study specifically used both hemoproteins and peroxides concentration range to mimic *in vivo* plasma conditions which ensue during acute or chronic vascular hemolysis. At this state of affairs, RH peroxidase-like activity, namely heme iron redox-shuttle (Fe^III^↔Fe^IV^), is limited and damage may result mostly from unstable hemin RH met-forms [6], [62].

The current study data indicate that metRH can be reduced to the more stable ferrous states with aid of endogenously HO-produced CO. The latter process (Fe^III^→Fe^II^) is analogous to the on going course of action provided to circulating Hb captured within red cells. Howether at more extreme pathological conditions the unstable hemin in ferric RH may be trapped by hemopexin [19]. It is only then hemopexin is exhausted and hemin leaks into LDL particles leading to irreversible damage [7].

It is well known in clinical biochemistry that an elevated COHb level is a marker for oxidative pathological processes such as in the case of hemolytic events [63], [64]. This elevation has been related to replacing oxygen in oxyHb with CO produced by induced HO. Our data support an as yet unrecognized medical benefit of carboxy hemoproteins formation, increasing vascular defense by CO-induced arrest of ferric Hb and elimination of peroxides.

## Materials and Methods

All participants provided informed, written consent; ethical approval for this study was obtained through the research ethics board of Tel-Aviv University.

### Materials

Myoglobin (equine), Hemin (from bovine red cells), Albumin (bovine serum, BSA), ethylene diamine tetraacetic acid (EDTA) and phenylmethylsulfonyl fluoride (PMSF) were purchased from Sigma-Aldrich, Israel. Hydrogen peroxide (H_2_O_2_) and potassium bromide (KBr) was from Merck, Darmstadt, Germany. PD-10 desalting columns were purchased from GE Healthcare UK Ltd. CM-52 and DE-52 celluloses were obtained from Whatman International, Maidstone, England. Ferricyanide was from Agan Chemical Manufacturers Ltd., Israel. Gases: Carbon monoxide (CO), at least 99.5%, was supplied by Gordon Gas and Chemicals Ltd., Israel; Nitrogen (N_2_, 99.9%) and Argon (Ar, 99.5%) were supplied by Israel Oxygen Centre.

### Hemoglobin isolation from fresh blood

Hemoglobin was purified from fresh human red blood cell lysates by ion-exchange chromatography using CM-52 cellulose followed by desalting dialysis. Since H_2_O_2_ was used in some reaction mixtures, Hb preparations were checked for lack of catalase by ferryl Hb formation in the presence of an equimolar amount of H_2_O_2_. Purified Hb was verified spectrophotometrically as oxyHb. MetHb was prepared from the oxyHb by oxidation with ferricyanide [65]. The reagent was removed usind PD-10 desalting column and globin-free hemin contaminants were removed by admixing the metHb solution with DE-52 cellulose [66]. Concentrations of all hemoproteins were measured spectrophotometrically and expressed in heme equivalents throughout this study.

### Low density lipoprotein (LDL) isolation

LDL was isolated from fresh human plasma (EDTA anticoagulated) of healthy donors by sequential ultracentrifugation as described [67], using Beckman Optima LE-80K ultracentrifuge (rotor Ti-70.1, 47,000 RPM). The obtained solution was passed through two sequential PD-10 columns to remove EDTA and other small molecules. LDL concentration was determined by the Lowry method using BSA as a standard and expressed as mg protein/ml [68].

### Oxidized LDL (oxLDL) preparation

Freshly isolated LDL was oxidized by copper according to the literature with some modifications [69]. Briefly, freshly prepared LDL (2–3 mg protein/ml) was incubated in the presence of CuSO_4_ (five fold molar protein concentration) for 2 hours in 37°C and immediately passed through two PD-10 columns.

### Aged LDL preparation

Freshly isolated LDL was sterilized by passing through 0.2 µM filters and further incubated for at least 10 weeks in 4°C [51]. Two standard parameters were used to determine LDL oxidation, conjugated diens and oxidation lag time. Conjugated dienes with the typical absorption maximum at 234 nm are used as a routine to follow LDL oxidation [52]. To follow the oxidation lag time 1 µM of hemin was used (stock solution was prepared in DMSO 80%). In the current study, as in previous, we used 268 nm as the undesired light scatter contribution is sharply reduced with decreased wavelength [15].

### Spectrophotometry

Spectral characteristics and time dependent changes spectral in reaction mixtures were monitored by single or repetitive (every 135 sec) scanning of light absorbance spectra at UV and/or visible wavelength range using a Contron – UVIKON_XL_ or thermostated GBC UV/VIS 920 spectrophotometer.

### Reactions conditions

All reactions were carried out in PBS pH 7.4 at 37°C. The appropriate gas atmosphere was reached as follows: the desired gas was bubbled into the media solutions in rubber septa sealed spectrophotometric cuvettes. Protein containing stock solutions were equilibrated a priori by the desired or nitrogen as neutral gas and were further diluted (by injection) into cuvettes up to 5% of volume. Under such conditions the gases concentration in the liquid phase reaches about 1 mM [43].

The following reactants were used at constant concentrations: hemoproteins - 10 µM, H_2_O_2_ - 30 µM (1∶ 250 dilution of stock), LDL - 100 µg protein/ml.
